# Detection and Antibiotic Treatment of *Mycoplasma arginini* Contamination in a Mouse Epithelial Cell Line Restore Normal Cell Physiology

**DOI:** 10.1155/2014/532105

**Published:** 2014-03-20

**Authors:** Brianna Boslett, Subhra Nag, Andrew Resnick

**Affiliations:** ^1^Department of Biology, Geology, and Environmental Science, Cleveland State University, 2121 Euclid Avenue, Cleveland, OH 44115, USA; ^2^Department of Physics, Cleveland State University, 2121 Euclid Avenue, Cleveland, OH 44115, USA; ^3^Center for Gene Regulation in Health and Disease, Cleveland State University, 2121 Euclid Avenue, Cleveland, OH 44115, USA

## Abstract

Mycoplasma contamination of cultured cell lines is difficult to detect by routine observation. Infected cells can display normal morphology and the slow growth rate of mycoplasma can delay detection for extended periods of time, compromising experimental results. Positive identification of mycoplasma typically requires cells to be either fixed and stained for DNA or processed with PCR. We present a method to detect mycoplasma using live-cell optical microscopy typically used for routine observation of cell cultures. Images of untreated mycoplasma-infected epithelial cells alongside images of infected cells treated with Plasmocin, a commercially available antibiotic targeted to mycoplasma, are shown. We found that optical imaging is an effective screening tool for detection of mycoplasma contamination. Importantly, we found that cells regained normal function after the contamination was cleared. In conclusion, we present a technique to diagnose probable mycoplasma infections in live cultures without fixation, resulting in faster response times and decreased loss of cell material.

## 1. Introduction

Mycoplasmas are small filterable bacteria, 0.1–0.8 microns in size, lack a cell wall, and are resistant to many antibiotics [1]. Mycoplasma contamination of cell cultures can progress undiscovered for long periods of time and is a significant problem in experimental research [2]. Typically, mycoplasma infections are verified via chapter 63 of the United States Pharmacopeia, chapter 2.6.7 of the European Pharmacopeia, or similar regulatory guidelines. While these culture methods are considered a “gold standard” of detection [3], these methods are more suitable for large-scale commercial lot release assays and, in the context of laboratory cell culture, may require sacrifice of suspected cells resulting in loss of experimental material and invalid experimental results if undetected contamination occurs during an experiment.

Recently, we suspected a mycoplasma contamination in an adherent epithelial cell line, a conditionally immortalized mouse renal cell line originally obtained by dissection of the cortical collecting duct from an Immortomouse [4]. Cells are seeded onto a suspended permeable membrane, and under sterile culture conditions, cells proliferate freely in “growth conditions” and are either passaged at 80% confluence or allowed to form a fully confluent monolayer. The confluent monolayer is then moved to “differentiation conditions” and over 10 days develops transepithelial resistance, directed sodium transport, and a “cobblestone” appearance. Once the epithelial tissue has gained function, the epithelial tissue is subjected to experimental protocols relevant to our research program (ciliary mediated mechanosensation). Regular electrophysiological measurements of the resistance and current are used to verify that the cell layer is intact and functional throughout experiments. Our laboratory does not prophylactically add antibiotics to culture media.

The cultures considered here ceased proliferating well before 80% confluence and displayed disorganized cell geometry and cells often detached from the permeable support. Culture media remained clear, and cells freshly drawn from frozen stocks did not form confluent monolayers. Repeated culture procedures for bacterial, yeast, and mold contamination were consistently negative. Mycoplasma contamination was initially suspected by fluorescence imaging of the nuclear stain DAPI applied to fixed cells, revealing extranuclear DNA. Surprisingly, initial efforts to obtain mycoplasma colonies by culture proved negative. The aims of this study were (1) to determine if mycoplasma contamination could be detected on living epithelial cell cultures using routine optical microscopy, (2) to identify the mycoplasma strain and determine if treatment with Plasmocin results in elimination of mycoplasma from our cell line, and (3) to determine if clearance of mycoplasma infection restores normal differentiated cell physiology in our cell line. We present evidence affirming all three goals.

## 2. Materials and Methods

### 2.1. Cultivation of Cell Line

Experiments were carried out with a mouse cell line derived from the cortical collecting duct (mCD 1296 (d)) of a heterozygous offspring of the Immortomouse (Charles River Laboratories, Wilmington, MA, USA). The Immortomouse carries as transgene a temperature-sensitive SV40 large T antigen under the control of an interferon-*γ* response element. Cells were maintained on collagen-coated Millicell-CM inserts (Millipore Corp, Billerica, MA) to promote a polarized epithelial phenotype. Cells were grown at 33°C, 5% CO_2_. The growth medium consisted of the following (final concentrations): Dulbecco's Modified Eagle Medium and Ham's F12 at a 1 : 1 ratio, 5 *μ*g/mL transferrin, 5 *μ*g/mL insulin, 10 ng/mL epithelial growth factor (EGF), 4 *μ*g/mL dexamethasone, 15 mM 4-(2-hydroxyethyl)-1-piperazineethanesulfonic acid (HEPES), 0.06% NaHCO_3_, 2 mM L-glutamine, 10 ng/mL mouse interferon-*γ*, 50 *μ*M ascorbic acid 2-phosphate, 20 nM selenium, and 5% fetal bovine serum (FBS). No prophylactic antibiotics were added to culture media. Confluent monolayers were maintained at 39°C, 5% CO_2_ to enhance differentiation. Under differentiation conditions, FBS, insulin, and interferon-*γ* were omitted from the apical medium and insulin, EGF, and interferon-*γ* omitted from the basal medium.

### 2.2. Antimycoplasma Antibiotic

Selected cell cultures maintained in growth conditions were treated with the compound preparation Plasmocin (InivoGen, San Diego, CA) at an initial concentration of 25 *μ*g/mL for 14 days. Plasmocin contains a mixture of a fluoroquinolone and a macrolide [5]. We found that passaging the cells at least once (1 : 1 dilution) during this treatment phase is required to fully eliminate the infection. After 14 days, Plasmocin concentration was reduced to 5 *μ*g/mL and applied for an additional 14 days. Plasmocin was then removed from the growth media and the cultures were allowed to recover for 14 days. No other antibiotics were added to the media, and cells were maintained in growth conditions during the entire 6-week treatment.

### 2.3. Electrophysiology

Cultures that completed Plasmocin treatment and formed confluent monolayers were moved to differentiation conditions. Transepithelial voltage and resistance measurements were performed using an Endohm chamber (World Precision Instruments, Sarasota, FL), connected to an Epithelial Voltohmmeter for transepithelial electrical resistance (TEER) measurements (EVOM, Millipore). The TEER measurement is highly sensitive to the health and physiological function of the epithelial monolayer. Cells were placed in the Endohm chamber for no more than 3 min, during which time the transepithelial voltage remained constant. The transepithelial voltage and resistance were converted to short-circuit current equivalent (Isc_eq_) assuming an ohmic relationship. More than 95% of Isc_eq_ was inhibited by 10 *μ*M apical amiloride and thus is considered to be proportional to the activity of epithelial sodium channels (ENaC) in the apical plasma membrane.

### 2.4. PlasmoTest

This assay uses an engineered cell line HEK-Blue (InvivoGen, modified Human Embryonic Kidney, HEK) to detect the presence of mycoplasma in culture media. HEK-Blue cells were expanded under normal culture conditions and then plated in 96-well plates for the assay. Media under test were added and the plate was placed within an incubator for 16–24 hours. The sensor cells detect the presence of mycoplasmas leading to a color change of the HEK-Blue detection medium. HEK-Blue cells recognize mycoplasmas through Toll-like Receptor 2 (TLR2), a pathogen recognition receptor. In the presence of mycoplasmas, TLR2 initiates a signaling cascade leading to the activation of NF-*κ*B and other transcription factors. These transcription factors induce the secretion of SEAP (secreted embryonic alkaline phosphatase) in the supernatant which is detected by the purple/blue coloration of the medium.

### 2.5. Immunocytochemistry

Fixation and immunocytochemistry were performed using standard techniques. Confluent monolayers were fixed in 4% paraformaldehyde for 15 min. After rinsing, the monolayers were permeabilized for 10 min with a solution of 0.1% Triton-X and 0.5% saponin in a blocking buffer containing 5% donkey serum, 5% goat serum, 1% bovine serum albumin (BSA), and 5% fetal bovine serum (FBS). The monolayers were then stained with a monoclonal mouse antibody against acetylated *α*-tubulin (Invitrogen, Carlsbad, CA) followed by an anti-mouse antibody labeled with AlexaFluor 488 (Invitrogen, Carlsbad, CA). Phalloidin-Texas Red was also applied to stain the actin cytoskeleton. The stained filter was cut out of the culture insert and transferred to a microscope slide, monolayer side up. The filter was mounted in VectaShield (Vector Labs, Burlingame, CA) with DAPI, a fluorescent molecule which strongly binds to A-T regions in DNA. A no. 1.5 coverslip was placed on top of the monolayer—if needed, small spacers were used to prevent the coverslip from compressing the monolayer. The slides were then sealed with nail polish and stored for imaging.

### 2.6. PCR

The Mycoplasma Plus PCR primer set (Agilent) was used to detect mycoplasma infections in our cell cultures. If the cells are infected with mycoplasma, the primers yield a single 874 bp amplification product, regardless of which species of mycoplasma is present in the sample. Restriction-fragment analysis of the amplification products using the restriction enzyme* Sau*3A I determined which species of mycoplasma was present in our samples. A species analysis was conducted by digesting a portion of the PCR sample, separating the restriction fragments using agarose gel electrophoresis and comparing the fragmentation pattern to the fingerprints that identify the mycoplasma species.

## 3. Results and Discussion

### 3.1. Mycoplasma Detection

We employed four independent methods to detect the presence of mycoplasma: Optical Microscopy, use of an engineered HEK cell line (PlasmoTest (InvivoGen, San Diego, CA)), DAPI nuclear staining, and PCR.

#### 3.1.1. Microscopy

**Figure 1:**
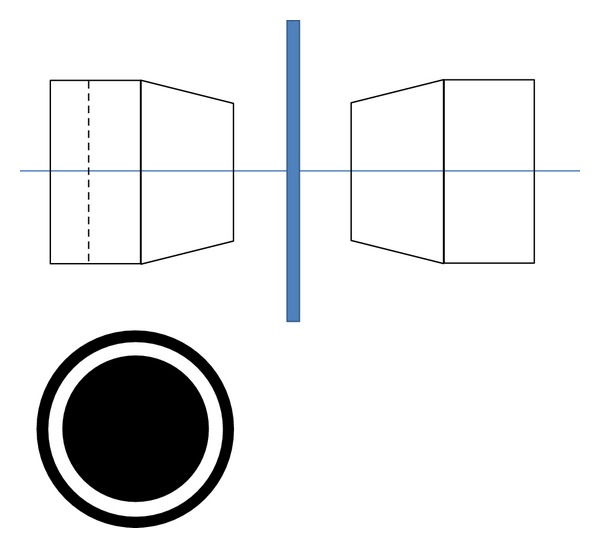
Schematic of illumination method. A schematic of the imaging method is shown here. The sample (middle) is Kohler illuminated using a high numerical aperture phase mask, resulting in annular illumination at a high angle of incidence. The objective lens, right, is not a phase objective but has a numerical aperture large enough to capture the unscattered annular illumination. We used a phase mask designed for a 40x/0.55 phase contrast objective and a 10x/0.25 objective lens to collect images.

While mycoplasmas are generally too small to be resolved with optical imaging, they are large enough to be detected due to light scattering. Using oblique illumination, a method similar to ultramicroscopy or darkfield imaging (Figure 1), mycoplasmas appear as bright punctate bodies attached to the cell membrane. While this method does not distinguish between mycoplasma and other subresolution contaminants, it can be performed on live cultures.

**Figure 2:**
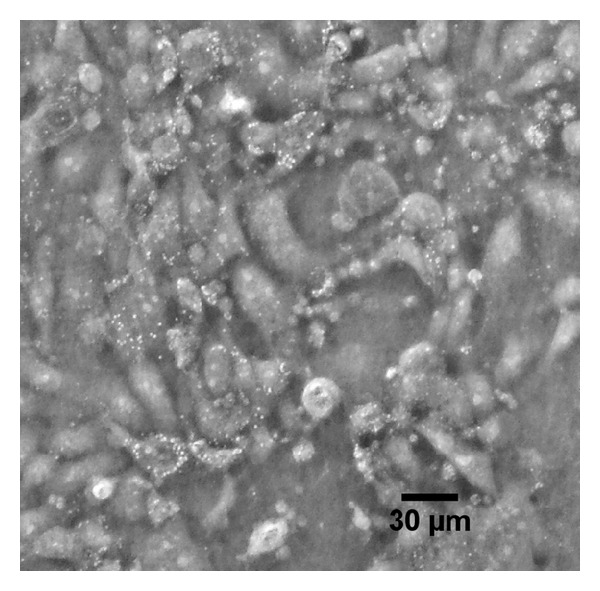
Example image of contaminated cells using oblique illumination. This image is of untreated cells, 18 d after plating. Plane of focus was set to the apical surface. Under normal viewing, cells appear healthy (no blebbing/apoptosis), while use of oblique illumination clearly shows the presence of bright punctate dots covering the apical membrane of many cells.

Images of cells were obtained under oblique illumination conditions (Figure 2). Oblique illumination displaces the illumination from centered on the optical axis to off-center and differs from darkfield illumination in that unscattered light enters the objective as well. It is important to note that use of oblique illumination introduces superperception effects but does not increase the resolving power: the Airy disk diameter is the same for brightfield and oblique illumination. In this way, the technique is similar to ultramicroscopy in that subresolution objects can be detected by imaging the scattered light against a darker background. Oblique illumination often produces asymmetric shading effects which we avoid by symmetrically illuminating the sample. We achieved oblique illumination through use of a condenser phase mask with a large diameter. The microscope used was a Nikon TMS inverted microscope outfitted with a 10x/0.25 objective and 40x condenser phase mask. The objective lens generates an Airy disk diameter of approximately 12 microns at the image plane, corresponding to optical resolution at the object plane of 1.2 microns. Mycoplasma and other subresolution particles are imaged as blur circles, bright punctate spots approximately 12 microns across (the Airy disc diameter), within the image. Blur circles can be seen by eye when using an eyepiece or may be directly imaged with a camera.

**Figure 3:**
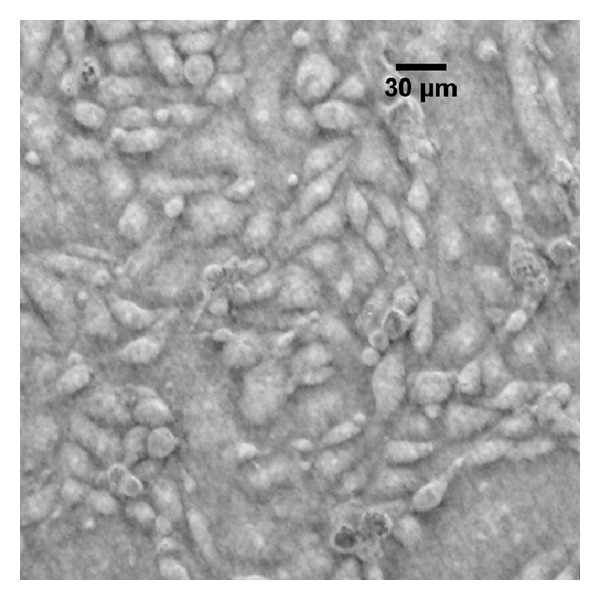
Example image of uncontaminated cells using oblique illumination. Plane of focus was set to the apical surface. Note the lack of punctate bodies.

**Figure 4:**
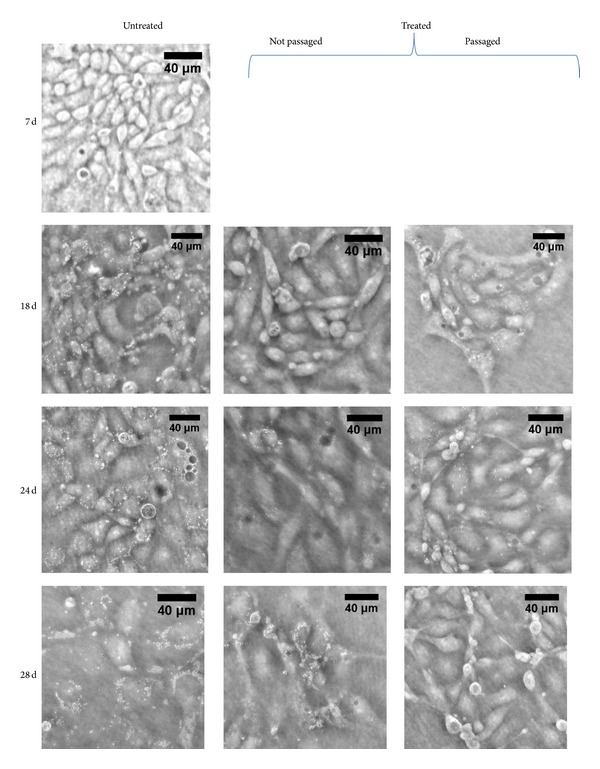
Image comparison during Plasmocin treatment. This series of images compare untreated cultures (left column) with Plasmocin-treated cultures (center and right columns) and also compare the effect of passaging cells during treatment (right column) with not passaging (center column). All images have the plane of focus set to the apical surface. Untreated cultures accumulate punctate bodies over time, while the treated cultures (Plasmocin treatment began on Day 7 after plating) instead show the elimination of punctate bodies. Passaging the cells during treatment appears to increase the rate of recovery, and after 3 weeks of Plasmocin treatment the cells that were passaged do not show any punctate bodies present.

Figures 2, 3, and 4 present optical images of live cultured cells before treatment, during treatment, and after treatment. Infected cells have a different morphology: cells aggregate in clumps rather than migrate and spread. We also show the effect of passaging the cells during Plasmocin treatment: cells that were passaged show significantly fewer punctate bodies, suggesting a more efficient clearance of contamination.

#### 3.1.2. PlasmoTest

The initial presence of mycoplasma was verified, and the successful eradication of mycoplasma was also verified. We conclude that, for our experimental system, PlasmoTest is a viable mycoplasma detection assay that can also be used without fixation of experimental material.

#### 3.1.3. DAPI Stain

**Figure 5:**
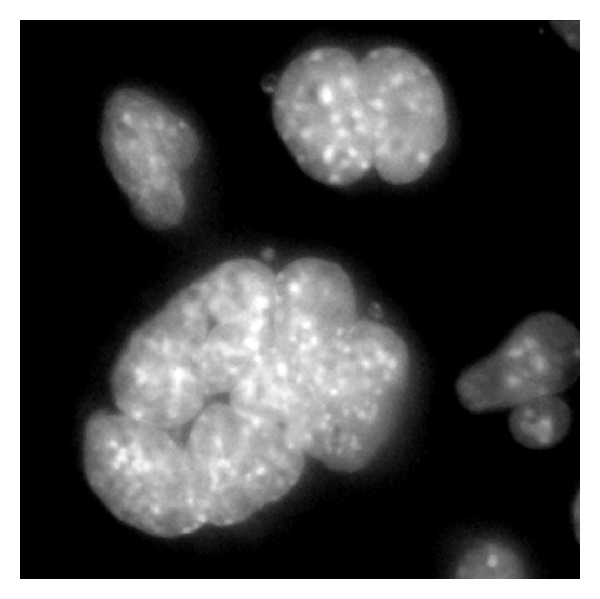
DAPI counterstained image of contaminated cells. This fluorescence image acquired with a 100x/1.47 immersion lens shows the presence of mycoplasma contamination. Evidence for mycoplasma contamination is given by the presence of large multinucleate cells, tightly clumped groups of cells, and the presence of small extranuclear DNA stains.

Cells were fixed with paraformaldehyde and mounted under standard conditions using VectaShield with DAPI. Samples were imaged with high magnification (100/1.47 objective lens) and mycoplasmas appear as extranuclear DNA.  Figure 5 presents an image showing mycoplasma infection.

#### 3.1.4. PCR

PCR amplification and restriction analysis resulted in identification of the mycoplasma species as* Mycoplasma arginini*. PCR also verified that the mycoplasma contamination was cleared by Plasmocin.

### 3.2. Posttreatment

Plasmocin successfully removed the contamination in 100% of the affected cultures that underwent passaging during antibiotic treatment, as verified by optical imaging, PlasmoTest, PCR, and DAPI staining. Over the course of this work, nearly 70 infected cultures drawn from 24 vials in frozen stock were examined. Positive and negative PlasmoTest results correlated exactly with PCR results; there were no false positives or false negatives. Thus, PlasmoTest is an effective detection method to verify the presence or absence of mycoplasma. Similarly, optical images of cells correlate with all other detection methods: the presence or absence of bright punctate bodies signals the presence or absence of mycoplasma. Thus, optical imaging can be an effective technique to screen cultures for mycoplasma contamination.

### 3.3. Electrophysiology

**Figure 6:**
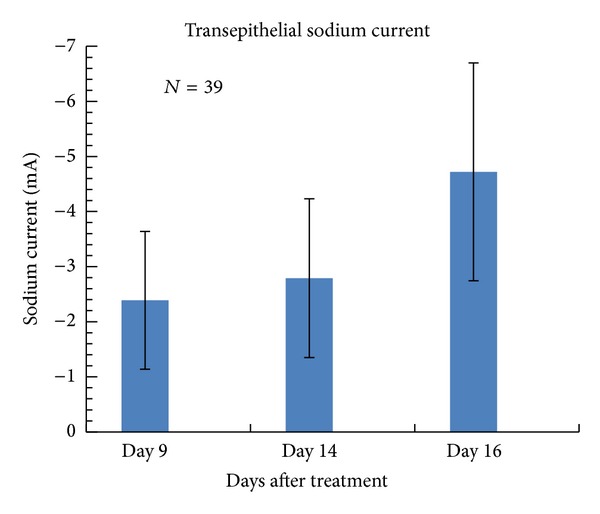
Electrophysiological data of treated cells. Transepithelial amiloride-sensitive sodium current measurements of Plasmocin-treated cells demonstrate full functional recovery after treatment. Note: we cannot show electrophysiological results of cells infected with mycoplasma since they could not survive the growth condition to enter differentiation.

In our cell line, transepithelial electrophysiological activity is a functional marker of differentiation. In healthy cultures, our cell line grows to confluence and upon differentiation forms an electrically resistant monolayer supporting directed salt and water transport [6]. However, contaminated cultures did not grow to confluence and thus could not form electrically resistant monolayers. Upon clearance of the contamination, cells again grew to confluence and formed an electrically confluent monolayer. Normal electrophysiological activity was obtained upon differentiation (Figure 6).

### 3.4. Immunocytochemistry

**Figure 7:**
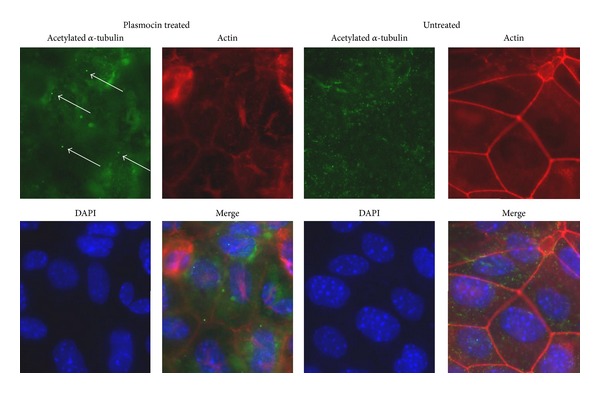
Comparison immunofluorescence images of treated and untreated cells. Cells were fixed and stained for acetylated *α*-tubulin, actin, and DAPI. Treated cells show the presence of a primary cilium (arrows). Infected cells show a disorganized microtubule cytoskeleton.

In our cell line, morphological differentiation is marked by the emergence of a primary cilium from the apical surface [7]. Cells were fixed and stained for acetylated *α*-tubulin, one of the proteins that form the ciliary axoneme. Posttreatment cells, upon differentiation, display normal cilia (Figure 7).

## 4. Conclusion

Mycoplasmas are smaller than most bacteria but large enough to scatter a significant amount of light which can be detected by light microscopy. Our images show the presence of punctate bodies attached to cell membranes when mycoplasma infection has occurred as verified by other mycoplasma screens and, conversely, the absence of bright punctate bodies is correlated with a mycoplasma-free cell culture; use of routine culture microscopy can thus trigger initial suspicion of mycoplasma, justifying more extensive detection methods such as PlasmoTest, DAPI, or PCR. The advantages optical microscopy provides are routine use (generally daily) and noninvasive nature, potentially resulting in rapid detection of contamination and a decrease in loss of experiments. While we have not applied this screen to a variety of mycoplasma strains, this simple screen should be generally applicable for adherent cell cultures and those mycoplasmas that remain attached to the cell membrane.

While the best way to eliminate mycoplasma contamination is to discard affected cultures, in some cases, for example, a valuable cell line, this is not a viable option. We have shown that our contaminated cultures can be successfully treated with Plasmocin and normal cell physiology resumes after the contamination has been cleared.
